# Aluminium hydroxide stabilised MnFe_2_O_4_ and Fe_3_O_4_ nanoparticles as dual-modality contrasts agent for MRI and PET imaging

**DOI:** 10.1016/j.biomaterials.2014.04.004

**Published:** 2014-07

**Authors:** Xianjin Cui, Salome Belo, Dirk Krüger, Yong Yan, Rafael T.M. de Rosales, Maite Jauregui-Osoro, Haitao Ye, Shi Su, Domokos Mathe, Noémi Kovács, Ildikó Horváth, Mariann Semjeni, Kavitha Sunassee, Krisztian Szigeti, Mark A. Green, Philip J. Blower

**Affiliations:** a King's College London, Division of Imaging Sciences and Biomedical Engineering, 4th Floor Lambeth Wing, St Thomas' Hospital, London SE1 7EH, UK; b School of Chemistry, Nottingham University, Nottingham NG7 2RD, UK; c School of Engineering and Applied Science, Aston University, Birmingham B4 7ET, UK; d CROmed Ltd., Baross u. 91-95, Budapest H-1047, Hungary; e King's College London, Department of Physics, Strand Campus, London WC2R 2LS, UK; f Department of Biophysics and Radiation Biology, Nanobiotechnology & In Vivo Imaging Center, Semmelweis University, IX. Tűzoltó u. 37-47, Budapest H-1094, Hungary; g King's College London, Division of Chemistry, Britannia House, 7 Trinity St, London SE1 1DB, UK

**Keywords:** Magnetic nanoparticles, PET, MR, Aluminium hydroxide, Dual-modal, ^18^F

## Abstract

Magnetic nanoparticles (NPs) MnFe_2_O_4_ and Fe_3_O_4_ were stabilised by depositing an Al(OH)_3_ layer *via* a hydrolysis process. The particles displayed excellent colloidal stability in water and a high affinity to [^18^F]-fluoride and bisphosphonate groups. A high radiolabeling efficiency, 97% for ^18^F-fluoride and 100% for ^64^Cu-bisphosphonate conjugate, was achieved by simply incubating NPs with radioactivity solution at room temperature for 5 min. The properties of particles were strongly dependant on the thickness and hardness of the Al(OH)_3_ layer which could in turn be controlled by the hydrolysis method. The application of these Al(OH)_3_ coated magnetic NPs in molecular imaging has been further explored. The results demonstrated that these NPs are potential candidates as dual modal probes for MR and PET. *In vivo* PET imaging showed a slow release of ^18^F from NPs, but no sign of efflux of ^64^Cu.

## 1 Introduction

Superparamagnetic nanoparticles (NPs) have been intensively investigated due to their potential applications in biosensors [1–3], targeted drug delivery [4–7], MRI [8,9] and localised hyperthermia induction [10,11]. An obstacle to application of these NPs is that they tend to aggregate and form larger secondary particles, in order to minimise their surface energy. Moreover, magnetic NPs are most often synthesised in organic solvents and coated with an organic layer of oleylamine or oleic acid rendering them soluble only in non-polar solvents. On the other hand, medical or bio-applications require colloidal stability and dispersibility in water and biological environments. Many methods have been developed to obtain stable colloids of magnetic NPs, reviewed by Laurent et al. [12]. Amongst them, coating with polyethyleneglycol (PEG) [8] or Dextran [13] has been widely used, as these hydrophilic and biocompatible materials not only provide a steric barrier against aggregation, but also make them hardly recognised by the macrophage-monocytic system [14]. To avoid desorption of the polymeric coating by heating or dilution, one or more functional groups, such as carbonate or phosphonate, are necessary to bind with the NPs. Such polymers, however, involve a complicated multi-step synthesis approach [8,15]. Therefore the use of an inorganic shell material that introduces stability, functionality and water-solubility is desirable.

Herein, we report a simple approach to stabilise magnetic NPs by coating them with an Al(OH)_3_ layer. The aluminium hydroxide coating was selected, due to its high affinity with fluoride anions [16] and bisphosphonate groups [17], which allow easy radiolabelling and functionalisation, and its biocompatibility as shown by its application in vaccine adjuvants [18].

## 2 Experimental section

### 2.1 Materials and general characterisation

All chemicals were used as purchased without further purification. Deionised water was obtained from an ELGA PureLab Option Q system. Bisphosphonate polyethyleneglycol (BP-PEG) polymers were synthesised in house according to published methods [8]. X-Ray powder diffraction (XRD) measurements were recorded at room temperature on a PANalytical X'Pert PRO diffractometer using Cu-K_α1_ radiation (*λ* = 1.540598 Å) at 40 kV, 40 mA, a scan speed of 0.02°/s and a step size of 0.026° in 2*θ*, at Nottingham University. X-Ray photoelectron spectra were recorded using a Thermo Fisher ESCALAB 250 X-ray Photoelectron Spectrometer with a hemispherical sector energy analyser at Aston University. Monochromatic Al K_α_ X-ray source was used at excitation energy of 15 kV and an emission current of 6 mA. The analyser pass energy of 20 eV with step size of 0.1 eV was used throughout the experiment. Transmission electron microscope (TEM) images were taken on a Tecnai FEI T20 at Centre for Ultrastructural Imaging, King's College London. Attenuated total reflectance infrared (ATR-IR or IR) spectra were recorded on a Perkin Elmer spectrum 100. Dynamic light scattering (DLS) experiments were carried out on Zetasizer Nano ZS from Malvern Instruments with a measure angle 175° and a 632.8 nm laser. Zeta potential for all samples was measured in neutral aqueous solution with a pH value ≈7.

### 2.2 Synthesis

#### 2.2.1 Synthesis of MnFe_2_O_4_ and Fe_3_O_4_

Magnetic NPs were obtained *via* a method reported previously [19,20]. Typically, 6 mmol 1,2-hexadecanediol was added to a 100 ml flask containing 20 ml phenyl ether, 5 ml oleylamine and 5 ml oleic acid at 120 °C, and the resultant solution was kept at this temperature under vacuum for over 30 min to remove water in the solvent. To this light yellow solution, 1 mmol Mn(acac)_2_ and 2 mmol Fe(acac)_3_ (or 2 mmol Fe(acac)_3_ for Fe_3_O_4_), was added under N_2_, and then temperature was increased to 270 °C at a rate of 10 °C/min with magnetic stirring. After 30 min, the flask was cooled to room temperature by removal from the hotplate. To precipitate out the NPs, 40 ml ethanol was added. The particles were collected by centrifugation (Jouan CR312, at a speed of 3000 rpm for 30 min) and washed with ethanol/hexane twice.

#### 2.2.2 Synthesis of MnFe_2_O_4_@Al(OH)_3_ (***1***)

MnFe_2_O_4_ (80 mg, 0.33 mmol) was dissolved in 30 ml diethyl ether by sonication for 20 min to form a dark brown solution, and then 10 ml of a diethyl ether solution containing AlCl_3_ (144 mg, 1 mmol) was added dropwise. The mixture was sonicated for 2 min before the addition of 500 μl water (27.8 mmol). The subsequent addition of 10 ml acetone led to a brown suspension. The product was collected by centrifugation and then dried in a stream of N_2_ to remove ether and acetone, and re-dispersed in water.

#### 2.2.3 Synthesis of Fe_3_O_4_@Al(OH)_3_ samples (***2***–***4***)

In the case of Fe_3_O_4_@Al(OH)_3_ (with a precursor molar ratio of Fe_3_O_4_ to AlCl_3_ of 1:3) (***4***), a faster uncontrolled hydrolysis method was used. Fe_3_O_4_ (82 mg, 0.33 mmol) was dissolved in 30 ml diethyl ether after sonication for 20 min to form a dark brown solution, and then 10 ml diethyl ether solution containing AlCl_3_ (144 mg, 1 mmol) was added dropwise. The mixture was sonicated for 2 min before the addition of 10 ml acetone leading to a brown suspension. The product was collected by centrifugation and then dried with a stream of N_2_ to remove ether and acetone, and re-dispersed in water. Corresponding amounts of AlCl_3_ were used with the same volume of Et_2_O to obtain Fe_3_O_4_@Al(OH)_3_ (1:1) (***2***) and Fe_3_O_4_@Al(OH)_3_ (1:2) (***3***) samples with various core–shell ratios.

#### 2.2.4 Filtration of MFe_2_O_4_@Al(OH)_3_ (M = Mn or Fe)

The Al(OH)_3_@MFe_2_O_4_ solution prepared as described in Section 2.2.2 (200 μl) was diluted with water (1 ml) to form a transparent brown solution, and then transferred to a 1 ml centrifuge tube with a filter inside (NanoSep, cut-off-molecular size, 30 K). Brown NPs were obtained on the filter by centrifugation at 5000 rpm for 20 min.

#### 2.2.5 Preparation of Fe_3_O_4_@Al(OH)_3_-BP-PEG(5K)

Bisphosphonate polyethyleneglycol (prepared as described elsewhere [8]) (5 mg) was added to the aqueous solution of Fe_3_O_4_@Al(OH)_3_ (5 ml, *ca*. 4 mg/ml), followed by a sonication treatment for 10 min.

### 2.3 Radiolabelling with ^18^F and radiochemical stability in water

^18^F labelling of MFe_2_O_4_@Al(OH)_3_ (M = Mn, or Fe, ***1***–***4***) was measured in triplicate at different concentrations. Typically, 50 μl aqueous [^18^F]sodium fluoride solution containing *ca*. 5 MBq radioactivity was added to a 450 μl solution of varying concentrations of MnFe_2_O_4_@Al(OH)_3_ in NanoSep with a cutoff size of 30k. After 10 min incubation with continuous shaking at room temperature, labelled NPs were separated by centrifugation at 5000 rpm (Eppendorf centrifuge 5424) for 20 min. The radioactivity of the supernatant and particles (on the filter) was measured separately using a gamma counter. The labelling efficiency was given by the following Equation (1):

(1) $$\text{Labelling}\;\text{efficiency}\;\left(\%\right)=\frac{\text{Activity}\;\text{of}\;\text{NPs}}{\text{Activity}\;\text{of}\;\text{NPs}+\text{Activity}\;\text{of}\;\text{supernatant}}\times 100\%$$

Triplicate samples of ^18^F labelled NPs were separated as described above. The NPs retained on the filter were re-suspended in deionised water in the inner NanoSep tube and then centrifuged at 5000 rpm for 20 min. This step was repeated three times. The percent binding retained after each washing step was calculated using equation (1). The correction for cumulative loss of label for the second and third washing steps was performed as exemplified by the following equation (2):

(2) CumulativeBinding=Activity%inNPs×Activity%inNPsprewash

### 2.4 Radiochemical stability of ^18^F-labelled ***1***, ***2***, ***3***, ***4*** in serum

Triplicate samples of labelled NPs were prepared on a NanoSep membrane as described above. The NPs retained in the filtrate were re-suspended in 25% serum in water (v/v), incubated at 37 °C for a period of up to 6 h, and then centrifuged at 10,000 rpm (Eppendorf centrifuge 5424) for 30 min. The cumulative binding was calculated using equation (2) as described previously.

### 2.5 Adsorption of non-radioactive ^19^F

5 mg NP ***1*** were dissolved in 5 ml freshly prepared NaF solution with concentrations of 0.01 mmol/L, 0.1 mmol/L, 1 mmol/L and 10 mmol/L. The suspensions of NPs were sonicated with the laboratory sonicator bath for 1 h, and then left overnight. The samples were centrifuged for 30 min at 3000 rpm (Jouan CR312) and 4 ml of supernatant was then withdrawn from each sample. The concentrations of fluoride anions in supernatant and corresponding particle-free NaF solution were measured with an Orion Star 214 bench-top meter with a fluoride combination electrode (from Fisher Scientific). Duplicate samples were prepared for each concentration. Adsorption percentage was obtained by dividing the concentration difference between the supernatant and the initial particle-free solution by the initial concentration.

### 2.6 [^18^F]-fluoride radiolabelling of washed Fe_3_O_4_@Al(OH)_3_ samples

500 μl of 1.34 mg/ml suspension of ***2*** in water (or 2 mg/ml ***3*** NPs, or 2.35 mg/ml ***4*** NPs) was placed in a NanoSep tube with omega membrane (molecular weight cutoff, 30 kDa). The tubes were centrifuged at 5000 rpm (Eppendorf centrifuge 5424) for 20 min, and then these NPs were re-dissolved in 450 μl water. 50 μl [^18^F]sodium fluoride (*ca*. 5 MBq) was added to these NPs solutions in the NanoSep tubes. After 10 min incubation by continuous shaking at room temperature, the tubes were centrifuged at 5000 rpm for 20 min. As described before, the activities in the filtrate and remaining on NPs (on the filter) were separately measured with a gamma counter, to produce a labelling efficiency for the 1st washed Fe_3_O_4_@Al(OH)_3_ samples. To measure the labelling efficiency for 2nd washed NPs, the washing step was repeat twice before incubation with ^18^F-fluoride radioactivity.

### 2.7 Radiolabelling of ***1*** with ^64^Cu

1 mg bis(dithiocarbamate) bisphosphonate (DTCBP) [15] was dissolved in 100 mm Na_2_CO_3_ buffer (pH 9). 200 μl of the above solution was added to 200 μl ^64^CuCl_2_ radioactivity (*ca*. 20 MBq) solution that was buffered to pH 5 with sodium acetate. It is essential to maintain the solution at neutral pH, since Al(OH)_3_ is not stable either in acidic or in basic solution. After 5 min, 200 μl 0.5 mg/ml MnFe_2_O_4_@Al(OH)_3_ solution containing 0.2 mg/ml PEG-5K was added and the mixture was incubated at room temperature for another 5 min. The radiolabelled NPs were isolated by filter centrifugation at speed of 5000 rpm for 15 min, using a Nanosep with a cutoff size of 30 K. There was no radioactivity observed in the filtrate, and all radioactivity remained on NPs in the filter. The ^64^Cu radiolabelled NPs were re-dissolved in 100 μl saline for injection.

### 2.8 *T*_1_, *T*_2_ and *T*_2_* relaxivity measurement

MR imaging of all particles was performed with a standard extremity flex coil on a clinical 3T Philips Achieva MRI scanner (Philips Healthcare, Best, The Netherlands). *T*_1_ mapping was obtained by using a 2D sequence that employed two non-selective inversion pulses with inversion times ranging from 20 to 2000 ms, followed by eight segmented readouts for eight individual images [21]. The two imaging trains resulted in a set of 16 images per slice with increasing inversion times (FOV = 200*200 mm, matrix = 200*179 mm, in-plane resolution = 1*1.12 mm, measured slice thickness = 3 mm, slices = 16, TR/TE = 3.2/1.6 ms, FA = 10°). *T*_2_ was determined with a 2D multi-spin-echo sequence (FOV = 200 × 200 mm, matrix = 200 × 200, measured slice thickness = 3 mm, ETL = 5, TE = 10 ms, TR = 725 ms, FA = 90°). The acquired imaging data were transferred to a computer running Matlab and analysed using an in-house Matlab tool to receive the relaxation times *T*_1_ and *T*_2_ for each NP concentration (in terms of [Fe] or [Fe] + [Mn]). Excel was used to plot the relaxation rates against concentration and the relaxivity (*i.e.* gradient of linear fit) determined from a least squares fit.

### 2.9 *In vivo* PET/MR imaging

A 6–7 weeks old female C57 black mouse with a weight of 20–21 g was used. Animal experiments were carried out at the Nanobiotechnology & In Vivo Imaging Center, Semmelweis University in Hungary, with permission from the local institutional animal ethics committee and in compliance with the relevant European Union and Hungarian regulations. PET/MRI images were recorded on a nanoScan (r) integrated PET/MRI system (Mediso, Budapest, Hungary), in which the MR is a preclinical 1T MRI scanner (M2, Aspect Imaging) with horizontal bore magnet, solenoid coil (diameter of 35 mm) and 450 mT/m gradients. Mice were anaesthetised with isoflurane and placed in prone position on the MRI bed. After the pre-contrast MR scan, 85 μl ^18^F-labelled (as described above, Section 2.3) NPs ***3*** solution in saline containing 0.95 MBq-fluoride radioactivity and *ca*. 60 μg Fe was injected *via* the tail vein. PET scanning was started immediately after injection and continued for 120 min. Acquisition took place in 1–5 coincidence mode with 5 ns coincidence window, 400–600 keV energy window, 94.7 mm scan range. A 3D expectation maximisation (3D EM) PET reconstruction algorithm (Mediso Tera-Tomo TM) was applied to produce PET images including corrections for attenuation and scatter, dead time, decay and randoms. After 8 iterations the reconstruction stopped resulting in images with 0.1 mm voxel size and time frames of 8 × 15 min. MR scanning was performed immediately after PET. The images of the two modalities were fused automatically.

### 2.10 *In vivo* PET/CT imaging

Two normal young C57BL/6 mice were used, at KCL in accordance with UK Research Councils' and Medical Research Charities' guidelines, under a UK Home Office licence. Mice were anaesthetised with isoflurane (Section 2.9) and 100 μl 0.5 mg/ml solution of ***1*** labelled with 6.98 MBq ^64^Cu (as described in Section…) in saline, containing 0.2 mg/ml PEG (5 K), was injected *via* tail vein. In the case of PET/CT imaging with ^18^F radiolabelled MnFe_2_O_4_@Al(OH)_3_-BP-PEG NPs (Sections 2.3 and 2.2.5), 105 μg NPs in 100 μl saline solution containing 4.48 MBq [^18^F]-fluoride radioactivity was injected. In the case of the control PET/CT imaging with “free ^64^Cu”, 50 μl ^64^CuCl_2_ solution buffered with sodium acetate (containing 5 MBq radioactivity) was injected intravenously *via* the tail vein. PET scanning was commenced immediately after injection of NPs using a NanoPET/CT scanner from Mediso, with PET acquisition time 120 min with a coincidence mode 1–5 and energy window 400–600 keV. CT scans were performed immediately after PET. Adjoint Monte Carlo was used for reconstruction, while the detector model and the number of iterations/subsets were LOR filter and 5/6, respectively.

## 3 Results and discussion

**Fig. 1:**
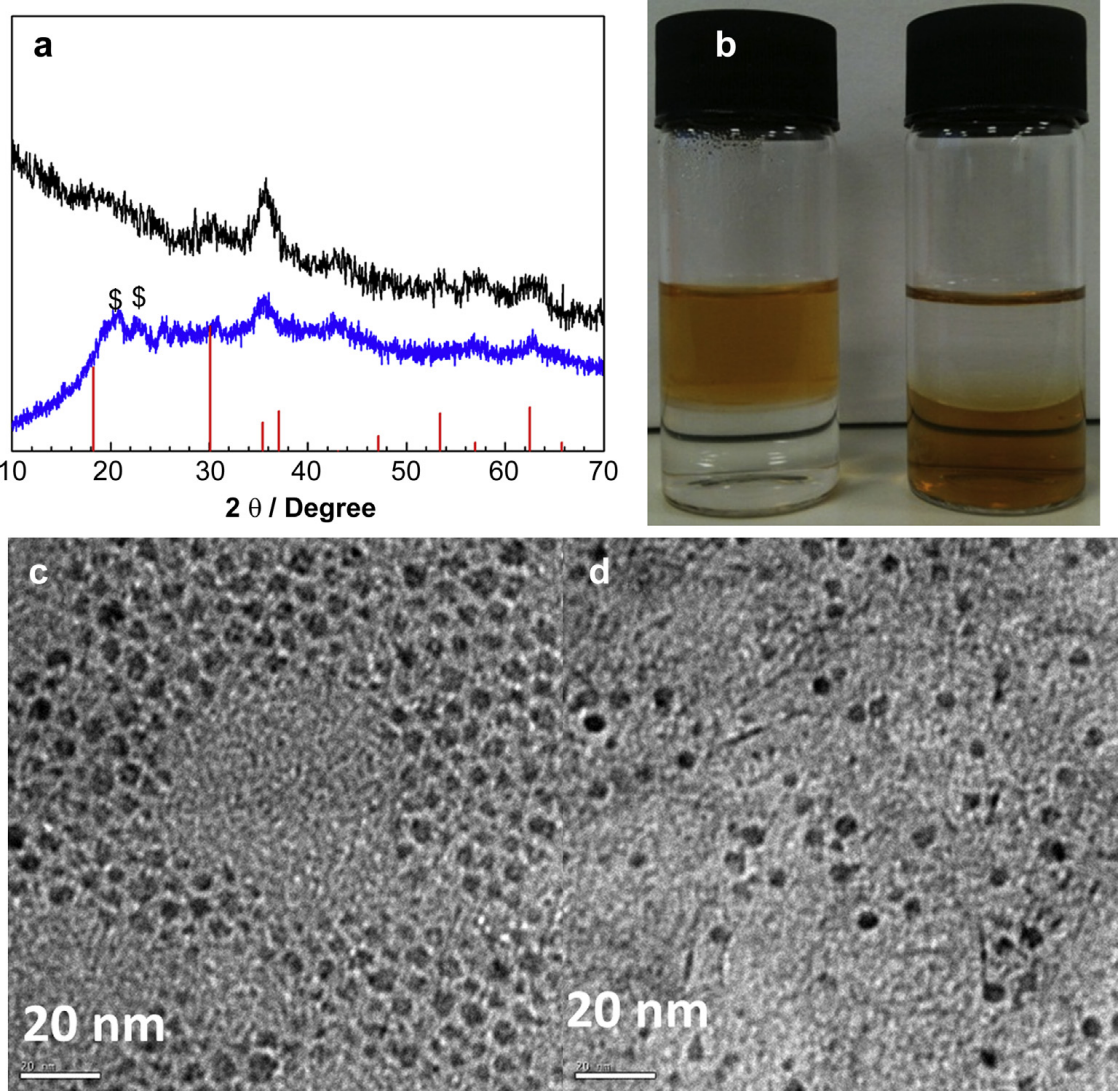
(a) XRD patterns of MnFe_2_O_4_ (black line) and MnFe_2_O_4_@Al(OH)_3_ (***1***) NPs (blue line). The red lines show the reference XRD pattern calculated from the published crystallographic data of Fe_3_O_4_[24]; $ represents the peak of Al(OH)_3_ (nordstrandite phase) [22]; (b) Photographs of MnFe_2_O_4_ (left) and ***1*** (right) NPs in a two-phase mixture of hexane (upper layer) and water (lower layer); (c) TEM images of MnFe_2_O_4_ NPs isolated from hexane; and (d) TEM image of ***1*** NPs isolated from water.

Typically, MnFe_2_O_4_@Al(OH)_3_ NPs (***1***) were obtained by adding a diethyl ether (Et_2_O) solution of AlCl_3_ to a Et_2_O solution of MnFe_2_O_4_ NPs, at the selected mole ratios, whilst stirring. After 10 min, the black mixture was treated with water (500 μl) to induce controlled hydrolysis and stirred for a further hour. The particles were precipitated out by the addition of 10 ml acetone, and then isolated by centrifugal filtration, washed with ethanol and re-dispersed in water. Fe_3_O_4_@Al(OH)_3_ samples with different Fe:Al ratios (***2***–***4***) were obtained *via* a quick hydrolysis process, where no water was added prior to the addition of acetone and AlCl_3_ was hydrolysed rapidly when the NPs were dispersed in water, rather than by addition of a small amount of water in Et_2_O. Two weak peaks around 21° in the XRD pattern appeared after coating and were associated with the nordstrandite phase of Al(OH)_3_ (Fig. 1a) [22]. The infrared spectrum of all Al(OH)_3_ coated samples showed the disappearance of adsorption peaks of C–H at 2845 cm^−1^ and 2950 cm^−1^ after coating with Al(OH)_3_, confirming that oleylamine had been removed, and the appearance of three absorption peaks at 842 cm^−1^ and 1645 cm^−1^ and a broad band from 3000 to 3500 cm^−1^, corresponding respectively to the Al–O stretching [23], the deformation vibration of water, and O–H stretching mode (Fig. S1). Nanoparticulate MnFe_2_O_4_ is soluble in hexane but insoluble in water due to the organic layer (oleylamine and oleic acid) on the surface. Once coated with Al(OH)_3_, the NPs become soluble in water but insoluble in hexane (Fig. 1b). All these features suggest a coating of Al(OH)_3_ replacing the oleylamine on the iron oxide NPs. Transmission electron microscopy (TEM), however, revealed no obvious difference size or morphology before and after coating with Al(OH)_3_ (Fig. 1c–d, Fig. S2). This could be attributed to the poorly crystalline and low-density nature of shell, indicated by the weak and broad diffraction peak on XRD pattern in Fig. 1a and Fig. S3.

X-Ray photoelectron spectroscopy (XPS) spectrum and inductively coupled plasma mass spectrometry (ICP-MS) both indicated that the content of Al in the shell increased with increasing ratios of AlCl_3_ to magnetic NPs (Table S1, Figs. S4 and 5). NPs with insufficient Al(OH)_3_, for example ***2*** tended to aggregate strongly in water, as indicated by TEM images (Fig. S2) and exhibited large hydrodynamic size (hydrodynamic diameter, *D*_h_) up to 400 nm as measured by dynamic light scattering (DLS) experiments (Table S2). This suggested the important role of Al(OH)_3_ in stabilising iron oxide NPs in water by converting the hydrophobic surface of oleylamine-coated Fe_3_O_4_ NPs into a hydrophilic surface, as well as offering a highly positive surface potential to protect them from aggregation. DLS experiments confirmed that NPs ***3*** exhibited a highly positive zeta potential up to +70 mV, and a small *D*_h_ of 21 nm, reduced from 43.8 nm for Fe_3_O_4_ in hexane (as measured by DLS). These coated NPs were stable in water with no obvious changes in *D*_h_ for over 12 months.

**Fig. 2:**
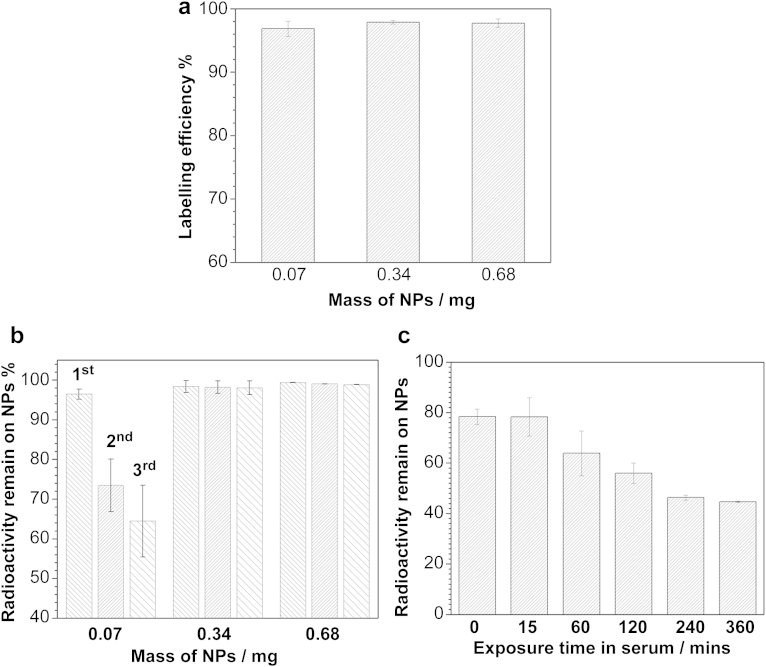
(a) No carrier added [^18^F]-fluoride radiolabelling of NPs ***1*** in water; (b) the amount of radioactivity remaining on ^18^F-fluoride labelled ***1*** NPs after washing with water 1, 2 and 3 times respectively; and (c) the amount of radioactivity remaining on NPs after incubation in human serum for different times (0–360 min). In the case of 0 min, the radiolabelled NPs were dissolved in serum in a 1 ml NanoSep with membrane, and then immediately isolated from serum by centrifugation.

Another benefit of the Al(OH)_3_ coating is its high affinity to fluoride ions and bisphosphonate groups [16,17], which allows a simple and easy approach for radiolabelling with [^18^F]-fluoride or metallic radionuclides conjugated with bisphosphonate. Indeed, a nearly 100% labelling efficiency (LE) was achieved by simply mixing a solution of NPs ***1*** with radioactive ^64^Cu(DTCBP)_2_ solution (Fig. 4a) [15] at room temperature for no more than 5 min, and no radioactivity was observed in the supernatant. Moreover, NPs ***1*** exhibited a high labelling efficiency (LE) with no-carrier-added [^18^F]-fluoride of up to 97% using as little as 10 μg NPs (Fig. 2). The adsorption of fluoride ions by Al(OH)_3_-coated NPs was further confirmed using a fluoride selective electrode, with cold NaF instead of tracer level radioactive ^18^F (Fig. S6). The binding capacity was measured to be up to 44.45 mg (fluoride)/g (NPs) (10 times higher than 4–7 mg/g observed for hydroxyapatite [16,25]). The kinetic stability of ^18^F binding to NPs (0.34 mg and 0.68 mg) was investigated in water and in serum. The results demonstrated that over 99.8% ^18^F remained on the NPs even after washing with water three times (Fig. 2b). However, the stability appeared to become diminished with a smaller sample of NPs (0.07 mg). This may be simply a result of mechanical losses due to manipulation of the very small sample. Studies on the dynamic stability in human serum indicated that there was a slow release of ^18^F from radiolabelled NPs ***1*** over a period of 4 h, with *ca*. 40% ^18^F remaining on NPs after 4 h incubation and no obvious further release of ^18^F-fluoride afterwards. The release of ^18^F into serum could be a combination of the dissociation of loosely bonded ^18^F from the surface, the substitution by other anions in serum, interaction with proteins in serum *via* hydrogen bonding or ion pairing, and the dissolution of a labile fraction of the Al(OH)_3_ layer.

Interestingly, initial results suggested that Fe_3_O_4_@Al(OH)_3_ samples (***2***–***4***), prepared by a fast, uncontrolled hydrolysis process, are much less efficient in radiolabelling with ^18^F than their analogues ***1*** prepared by controlled hydrolysis (Fig. S7). Moreover, NPs coated with a thicker Al(OH)_3_ layer, for example NPs ***3*** and ***4***, showed a worse LE, less than 10%, but higher colloidal stability. NPs ***2*** have a thinner shell and correspondingly lower colloidal stability. These phenomena lead to the hypothesis that a quick hydrolysis with large amount of water resulted in an unstable Al(OH)_3_ layer on the NPs (***2***–***4***) whereas a slow hydrolysis with a small amount of water in Et_2_O led to a stable layer (***1***). An external unstable Al(OH)_3_ layer would be washed into the supernatant during the separation process, resulting in a low value of LE. By monitoring the Al concentration in the supernatant after washing and comparing to the initial solution by ICP-MS, we found that almost half of the aluminium was washed out at the first wash for samples ***3*** and ***4*** which were synthesised by the quick hydrolysis process. The aluminium remaining on the NPs after washing was stable since no Al was detected in the supernatant after further washing (Table S3). Correspondingly, these NPs ***2***–***4*** displayed a high affinity to [^18^F]-fluoride after washing, of up to 94.9%. Only trace amounts of Al were detected in the supernatant of ***1***, which suggested a stable layer of Al(OH)_3_ consistent with the excellent radiolabelling results above.

**Fig. 3:**
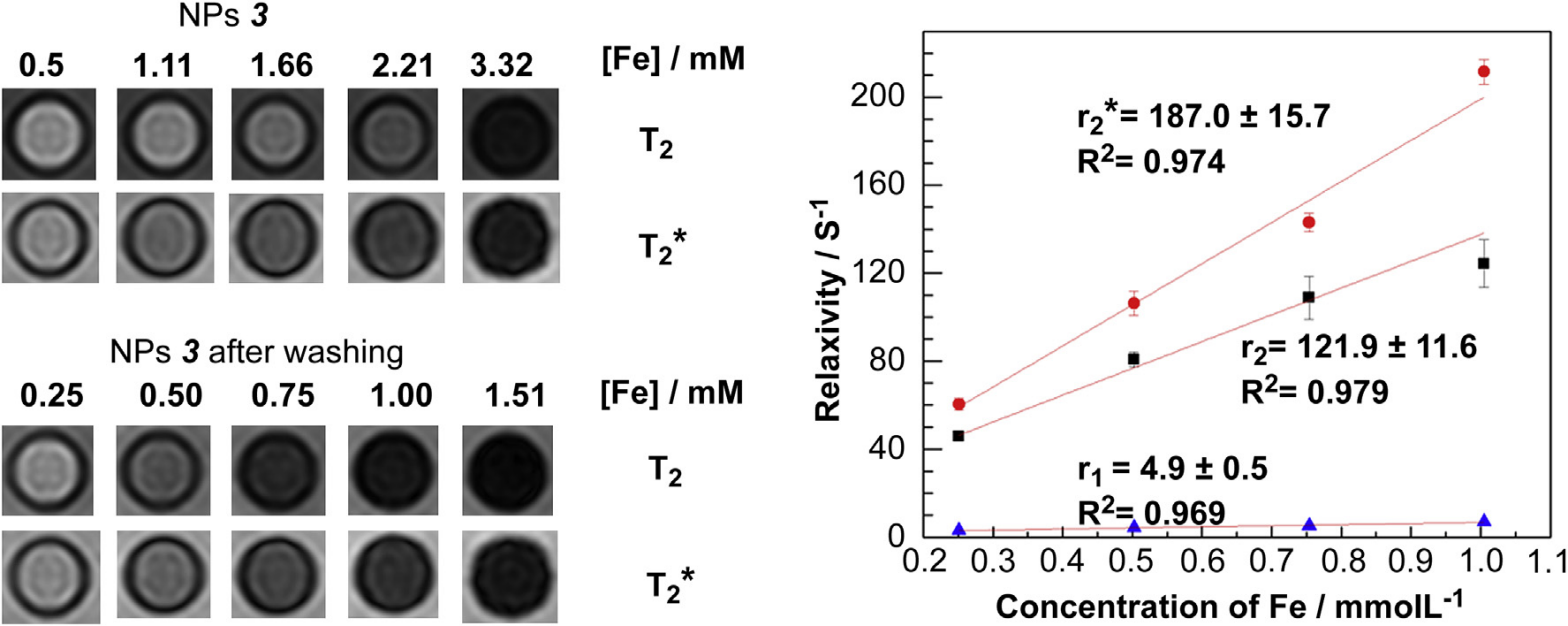
Left: *T*_2_ and *T*_2_* weighted MR images of aqueous solutions of the NPs ***3*** (upper series) or the NPs ***3*** after being washed with water using NanoSep (lower series); right: curves of relaxivity against concentration at 3T (red circles, *r*_2_*; black squares, *r*_2_; blue triangles, *r*_1_). The concentration of Fe was measured by ICP-MS.

As expected, these Al(OH)_3_-coated NPs displayed essentially the magnetic properties of the cores and were active as contrast agents in MR imaging, showing a darkening contrast on the *T*_2_ or *T*_2_* weighted MR images of solutions of NPs as a result of shortening transverse relaxation time of water molecules (Fig. 3). The transverse relaxivity property (*r*_2_) of the NPs strongly depends on the shell thickness, weakening dramatically as the Al(OH)_3_ shell thickness increases (***3*** and ***4***), consistent with previous reports that relaxivity is proportional to the volume fraction of magnetic materials [26]. Fe_3_O_4_@Al(OH)_3_ samples ***3*** and ***4*** displayed higher relaxivities (*r*_1_ and *r*_2_) after washing off the unstable layer; for example, *r*_2_ was improved from 81.6 to 121.9 mm^−1^ s^−1^ for NPs ***3***, and from 60.5 to 116.6 mm^−1^ s^−1^ for NPs ***4*** at 3T magnetic field (Fig. 3, Table S2). For the samples with a stable layer (***1***), no obvious improvement was observed on the relaxivity properties after washing.

**Fig. 4:**
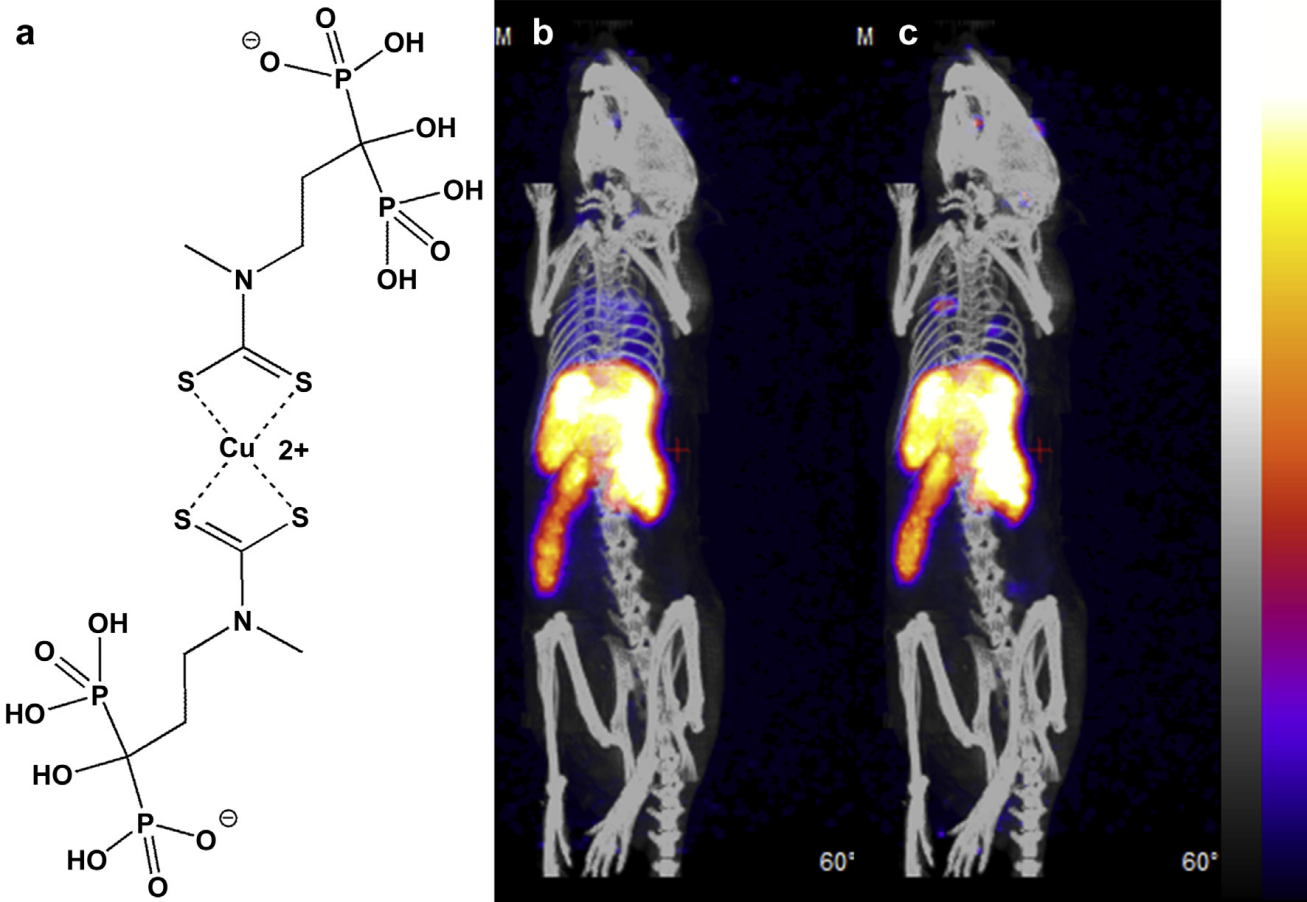
Structure of ^64^Cu(DTCBP)_2_, the bisphosphonate derivative used to bind ^64^Cu to NPs (a); and *in vivo* PET/CT images (maximum intensity projection) of a normal young C57BL/6 mouse after intravenous injection with ^64^Cu radiolabelled ***1***, showing dynamic biodistribution of NPs, 0–15 min (b) and 105–120 min (c).

*In vivo* PET/CT and PET/MR imaging results showed that both ***1*** and ***3*** labelled with [^18^F]-fluoride exhibited a quick uptake, seen by PET imaging, in the spleen and liver after intravenous injection *via* tail vein, despite their small hydrodynamic size of 21 nm in saline solution. Accumulation of NPs in the spleen and liver was evident also by MR, in a significant darkening contrast in the corresponding areas on MR images in Fig. S8. The combined images show that the magnetic cores and the radioactivity co-localise in the early period after injection but separate with time. Due to the unstable aluminium hydroxide shell, [^18^F]-fluoride radioactivity was gradually released from NPs ***3*** *in vivo*, resulting in a significant bone uptake increasing with time. Consistent with *in vitro* studies presented above, ***1*** NPs showed a better *in vivo* stability and slower, but still significant, release of [^18^F]-fluoride radioactivity (Fig. S9). By contrast, intravenously injected free [^18^F]-fluoride, without NPs, is immediately accumulated in bone and not in liver and spleen. PET/CT imaging a normal mouse with ***1***-^64^Cu(DTCBP)_2_ showed a similar biodistribution to that of ^18^F radiolabeled NPs (Fig. 4). All intravenously administered NPs were taken up by the spleen and liver within 2 h post-injection, and showed no sign of efflux of radiolabel from these organs, in contrast to the ^18^F-labelled particles. By comparison, PET/CT using ionic ^64^Cu (^64^CuCl_2_, Fig. S10) showed uptake dominated by liver and kidneys but not spleen. This confirms that ^64^Cu radioactivity attached to NPs *via* bisphosphonate groups co-localises with the magnetic cores and is not rapidly detached from the NPs. The quick clearance of ***1*** NPs by the liver and spleen was not unexpected, as the *in vivo* behaviour is determined not only by their hydrodynamic size but also by surface properties (surface chemistry and potential) [27,28]. Generally, intravenously administered NPs over 100 nm are readily cleared by the reticuloendothelial system (RES) through opsonisation, whilst small particles (10–100 nm) tend to stay in the blood pool longer [27]. Thus, although their hydrodynamic size as measured in saline or in water was sufficiently small, to achieve stealth features, the Al(OH)_3_-coated NPs needed further surface modification to neutralise the surface potential. We found that in this case, polymers with anionic functional groups bound to the NPs *via* bisphosphonate groups, such as bisphosphonate polyethyleneglycol (BP-PEG), could be used to modulate the surface potential of particles (Fig. S11), and protect them from opsonisation and aggregation in serum.

## 4 Conclusions

In summary, we have presented a simple approach to convert hydrophobic iron oxide-based magnetic NPs into hydrophilic particles stabilised by an Al(OH)_3_ shell. The features of this system, including high efficiency on ^18^F or ^64^Cu labelling, excellent colloidal stability, small hydrodynamic size, good transverse relaxivity and controllable surface potential, suggest that materials based on Fe_3_O_4_@Al(OH)_3_ have potential applications as bimodal contrast agents in PET/MRI imaging. A slow release of ^18^F from NPs was observed *in vivo*, whereas PET imaging with ^64^Cu radiolabelled NPs showed no loss of radioactivity from the initially targeted organs (liver, spleen). The ability to derivatise the surface with radiolabels and bisphosphonate groups suggests applications in molecular imaging. Barriers to *in vivo* use due to toxicity should be low, because of the established use of Al(OH)_3_ as adjuvants in vaccines. The high affinity to bisphosphonate groups for Al(OH)_3_ allows us to conjugate these NPs with a range of imaging and therapeutic radionuclides which may be used in conjunction with magnetic imaging and therapy.
