# Supplementary material for: Aluminium hydroxide stabilised MnFe2O4 and Fe3O4 nanoparticles as dual-modality contrasts agent for MRI and PET imaging
Source: Biomaterials. 2014 Jul;35(22):5840–6. doi: 10.1016/j.biomaterials.2014.04.004 (PMC4026944; doi:10.1016/j.biomaterials.2014.04.004)
Supplement: Supplementary file 1 [file mmc1.docx]

**Supplementary Data**

**Table S1:** Compositional results (ICP-MS) of Al(OH)_3_ coated NPs without treatment.

| **Samples**  (***label***) | **Precursors ratio**  MFe_2_O_4_ to AlCl_3_  (M = Mn or Fe) | **Elements to detect (mmo/L)** | | | **Core-shell ratio**  MFe_2_O_4_ to Al(OH)_3_  (M = Mn or Fe) |
| --- | --- | --- | --- | --- | --- |
|  |  | Fe | Mn | Al |  |
| MnFe_2_O_4_@Al(OH)_3_  (***1***) | 1: 3 | 10.29 | 0.98 | 26.79 | 1: 7.3 |
| Fe_3_O_4_@Al(OH)_3_ (1:1)  (***2***) | 1: 1 | 7.79 | n/a | 3.05 | 1: 1.2 |
| Fe_3_O_4_@Al(OH)_3_ (1:2)  (***3***) | 1: 2 | 7.92 | n/a | 8.22 | 1 : 3.1 |
| Fe_3_O_4_@Al(OH)_3_ (1:3)  (***4***) | 1: 3 | 7.98 | n/a | 11.41 | 1 : 4.3 |

**Table S2.** DLS results for alumina coated samples before and after filtration

| **Sample** | **D_h_ / nm** | | **Zeta potential / Mv** | | ***r_1_*** **/ mM^-1^s^-1^** | | ***r_2_*** **/ mM^-1^s^-1^** | |
| --- | --- | --- | --- | --- | --- | --- | --- | --- |
|  | **Before** | **After** | **Before** | **After** | **before** | **after** | **before** | **After** |
| ***1*** | 21.0 | 50.7 | +72.3 | +55.9 | 1.47 | 0.65 | 21.4 | 18.0 |
| ***2*** | 18.2 | 50.8 | +70 | +52.5 | 1.65 | 5.36 | 60.5 | 116.6 |
| ***3*** | 21.0 | 49.8 | +49.8 | +38.9 | 3.54 | 3.7 | 81.6 | 121.9 |
| ***4*** | 396.1 | 458.0 | +27.0 | +18.4 | n/a | n/a | n/a | n/a |

**Table S3.** ICP-MS analysis of Al and Fe in pre-wash NPs colloids and the supernatant after wash.

|  | | **Pre-wash**  **NPs colloid** | **1^st^ wash**  **supernatant** | **2^nd^ wash**  **supernatant** | **3^rd^ wash**  **Supernatant** |
| --- | --- | --- | --- | --- | --- |
| ***2*** | [Fe] mM | 7.79 | Not detected | Not detected | Not detected |
|  | [Al] mM | 3.05 | Not detected | Not detected | Not detected |
| ***3*** | [Fe] mM | 7.92 | 0.13 | Not detected | Not detected |
|  | [Al] mM | 8.22 | 2.96 | 0.07 | Not detected |
| ***4*** | [Fe] mM | 7.98 | 0.44 | 0.01 | Not detected |
|  | [Al] mM | 11.04 | 5.55 | 0.15 | Not detected |

**
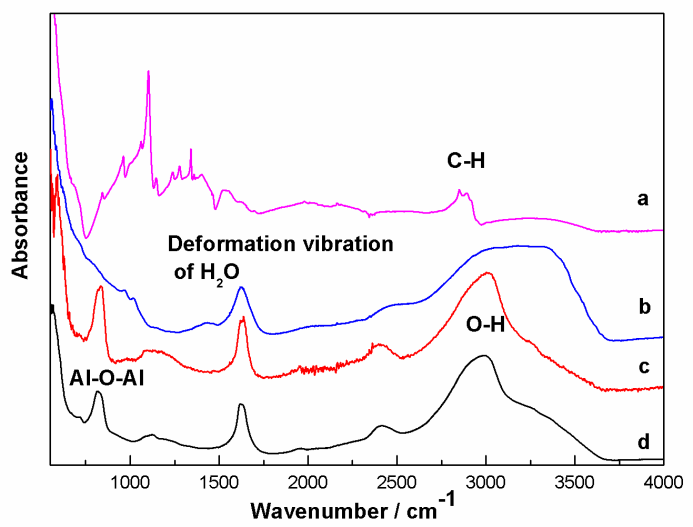
**

**Figure S1.** IR spectrum evolution of Fe_3_O_4_@Al(OH)_3_ samples: **a)** as-synthesised Fe_3_O_4_ sample, **b)** NP ***2***, **c)** NP ***3***, and **d)** NP ***4***

**
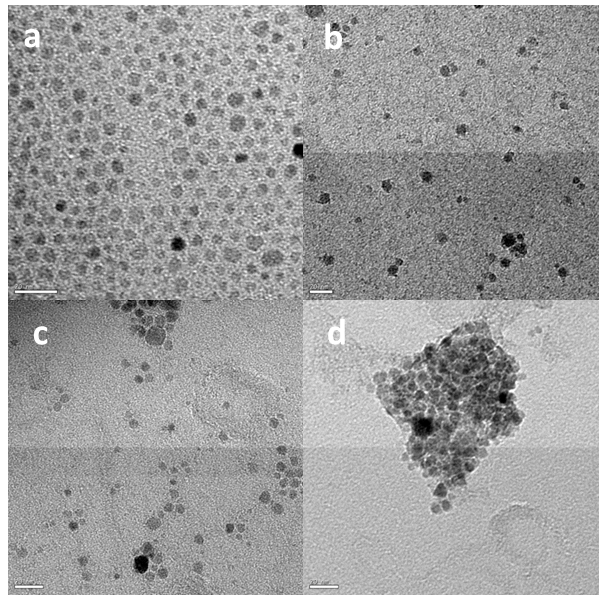
**

**Figure S2.** TEM images of Fe_3_O_4_ NPs and Fe_3_O_4_@Al(OH)_3_ NPs. **a)** Fe_3_O_4_ NPs prepared from hexane solution, **b)** NPs ***4*** prepared from aqueous solution, **c)** NPs ***3*** prepared from aqueous solution, and **d)** NPs ***2*** prepared from aqueous solution.


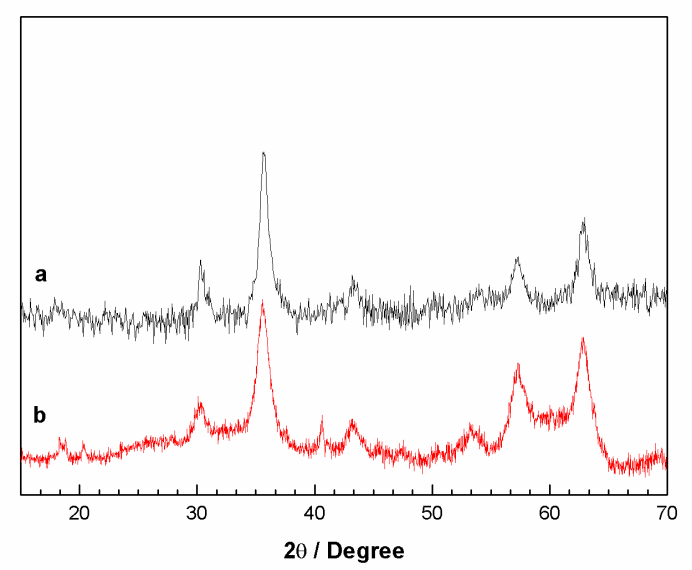


**Figure S3.** XRD pattern of samples: **a)** NP Fe_3_O_4_ and **b)** NP ***2***

**
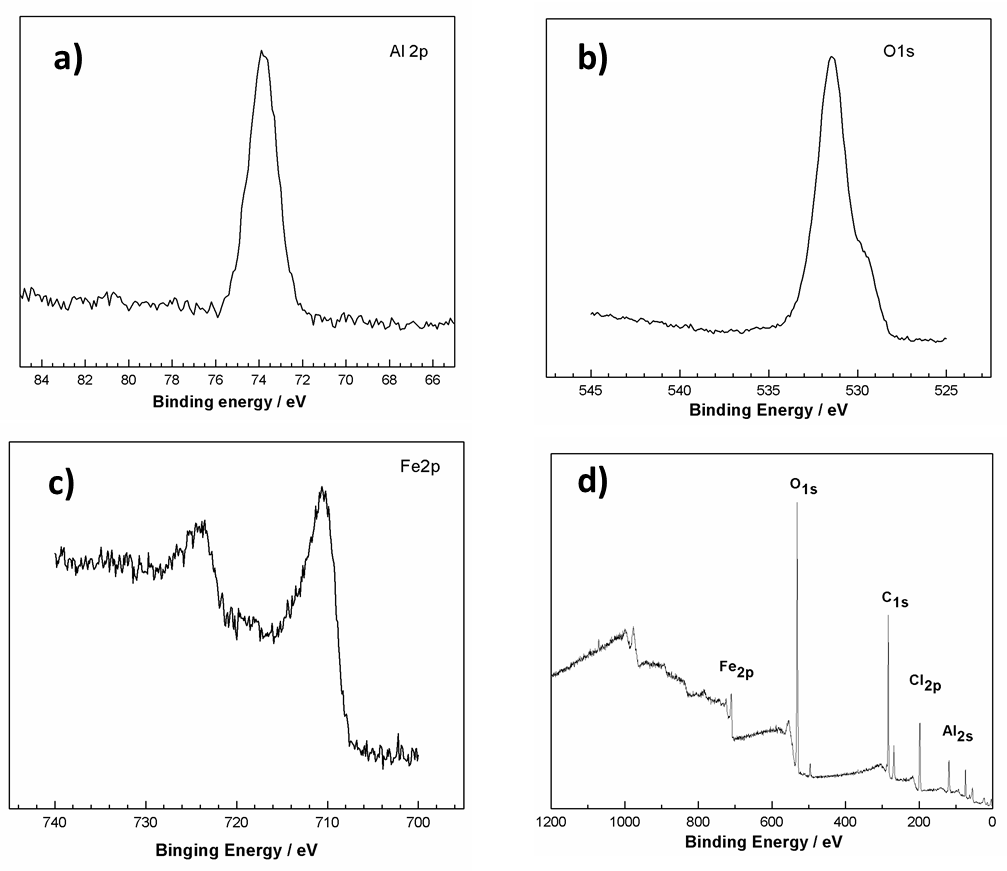
**

**Figure S4.** XPS spectrum of sample ***4***. **a)** Al_2p_ level data, **b)** O_1s_ level data, **c)** Fe_2p_ level data, and **d)** full scan of XPS spectra.

**
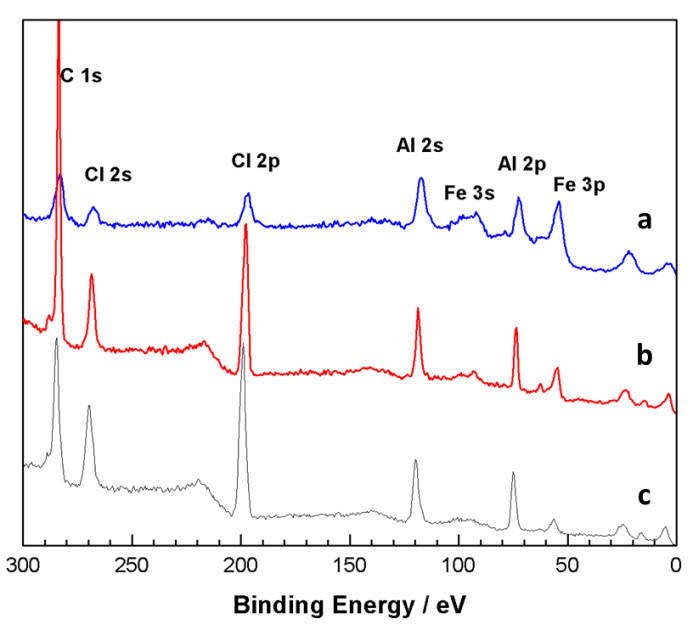
**

**Figure S5.** XPS spectra comparison of Fe_3_O_4_@Al(OH)_3_ samples (***2***- ***4***) with different core-shell ratio: **a)** NP ***2*,** 1:1; **b)** NP ***3***, 1:2; and **c)** NP ***4***, 1:3.

**
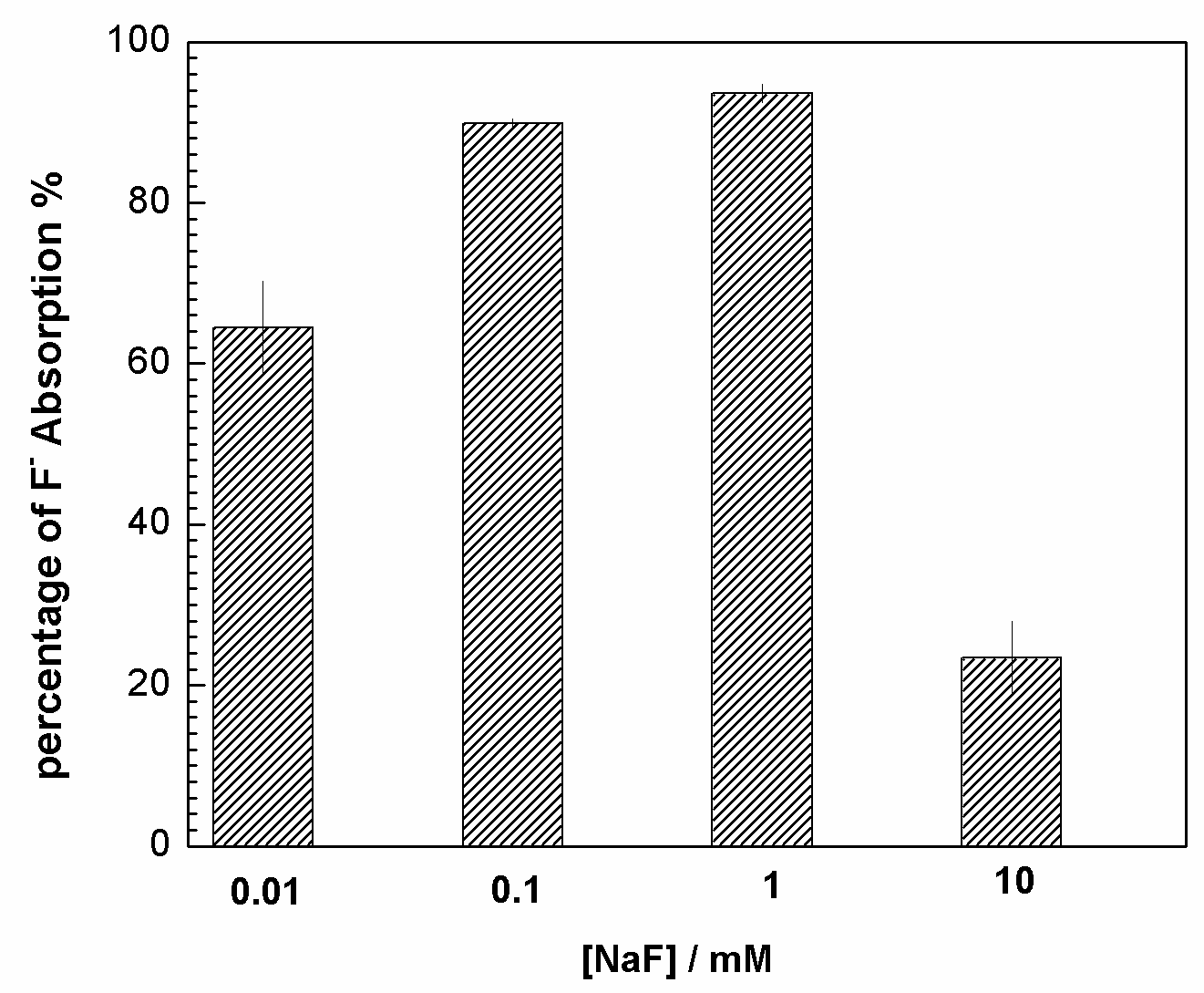
**

**Figure S6.** Adsorption of non-radioactive ^19^F^-^ by 5 mg ***1*** NPs in 5 ml NaF solution of different conditions, monitored by fluoride ion selective electrode.

**
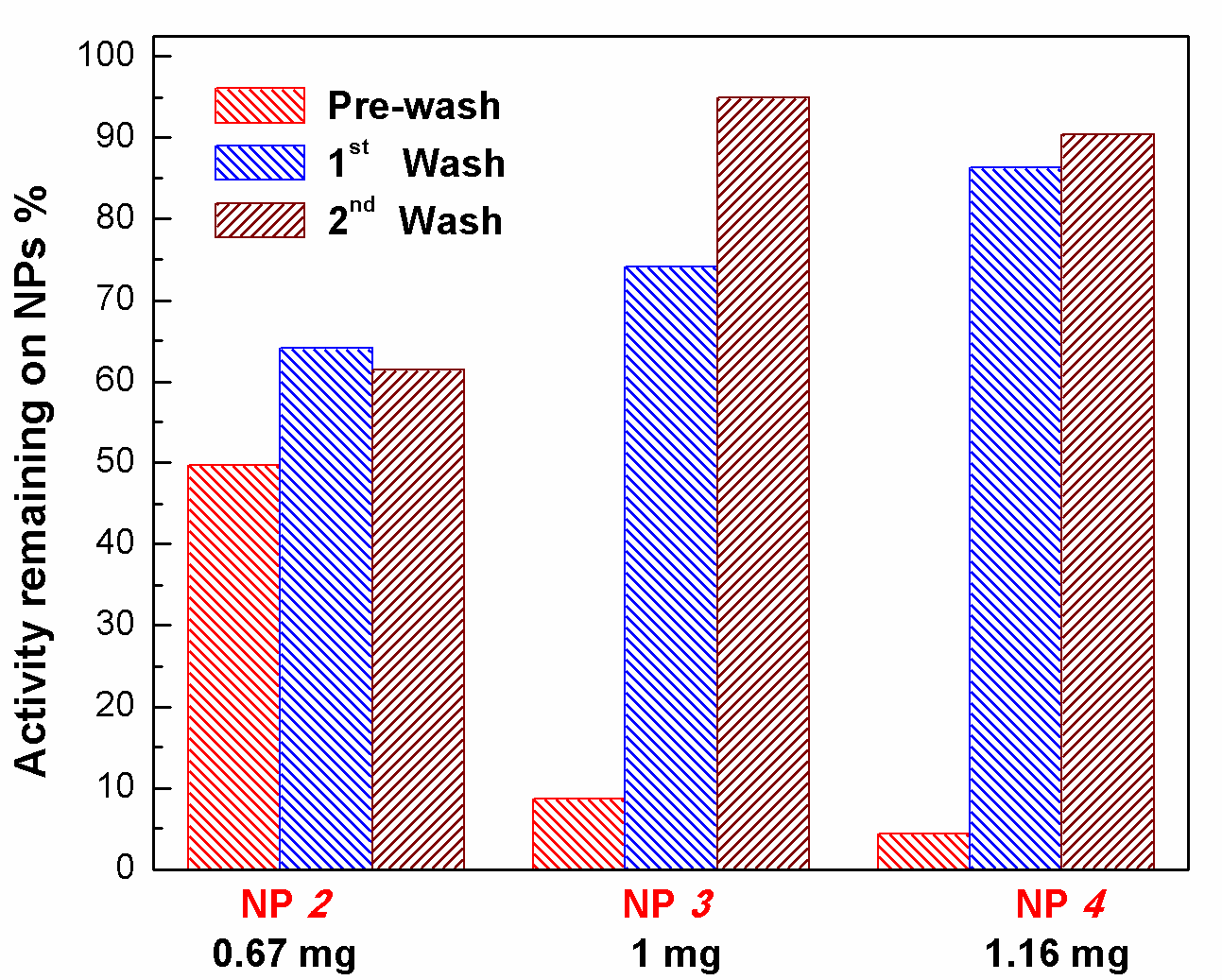
**

**Figure S7.** ^18^F-fluoride radio labelling of Fe_3_O_4_@Al(OH)_3_ NPs (***2***-***4***) varying the core-shell ratio. Pre-wash results showed that all three samples exhibited a low labelling efficiency, especially ***3*** and ***4***. A much higher radiolabelling efficiency were achieved after removal of unstable Al(OH)_3_ layer by washing with water 1 or 2 times.

**
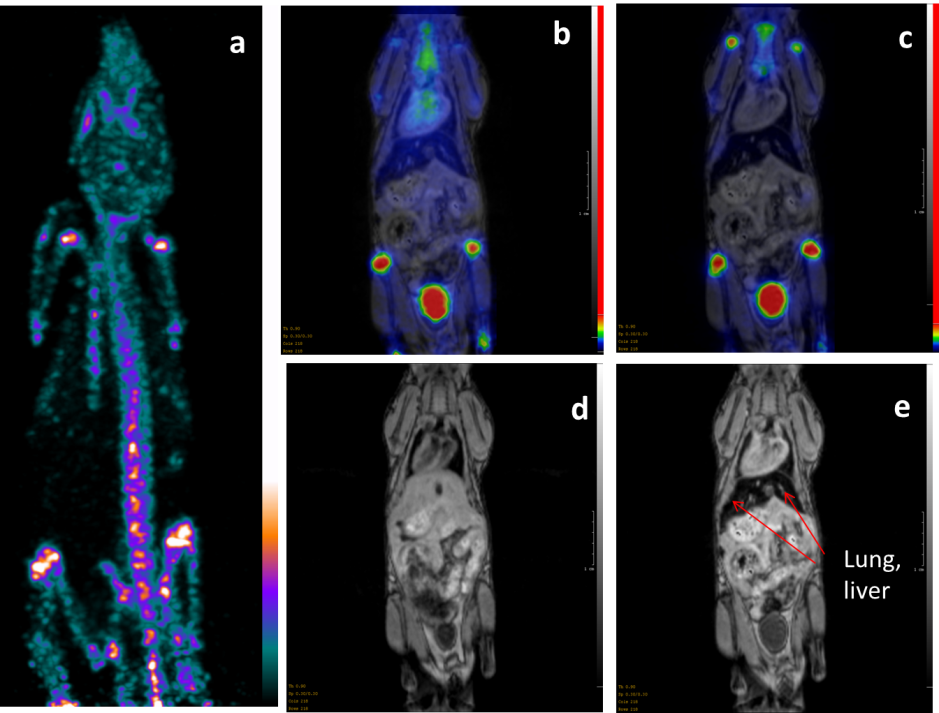
**

**Figure S8.** *In vivo* PET/MRI images of a normal young C57BL/6 mouse using ^18^F radiolabelled ***3***: **(a)** whole body PET image showing distribution of ^18^F 30 minutes post injection (maximum intensity projection, mice in prone position); **(b)** PET/MRI fused image (coronal section, 0-15 minutes); **(c)** PET/MRI fused image (coronal section, 105-120 minutes); **(d)** MR image prior to the injection of NPs, and **(e)** MR image post the injection of NPs, showing a darkening contrast at lung and live area. Due to the unstable Al(OH)_3_ shell, ^18^F-fluoride radioactivity was released from magnetic NPs ***3*** within 15 minutes and localised in bone.

**
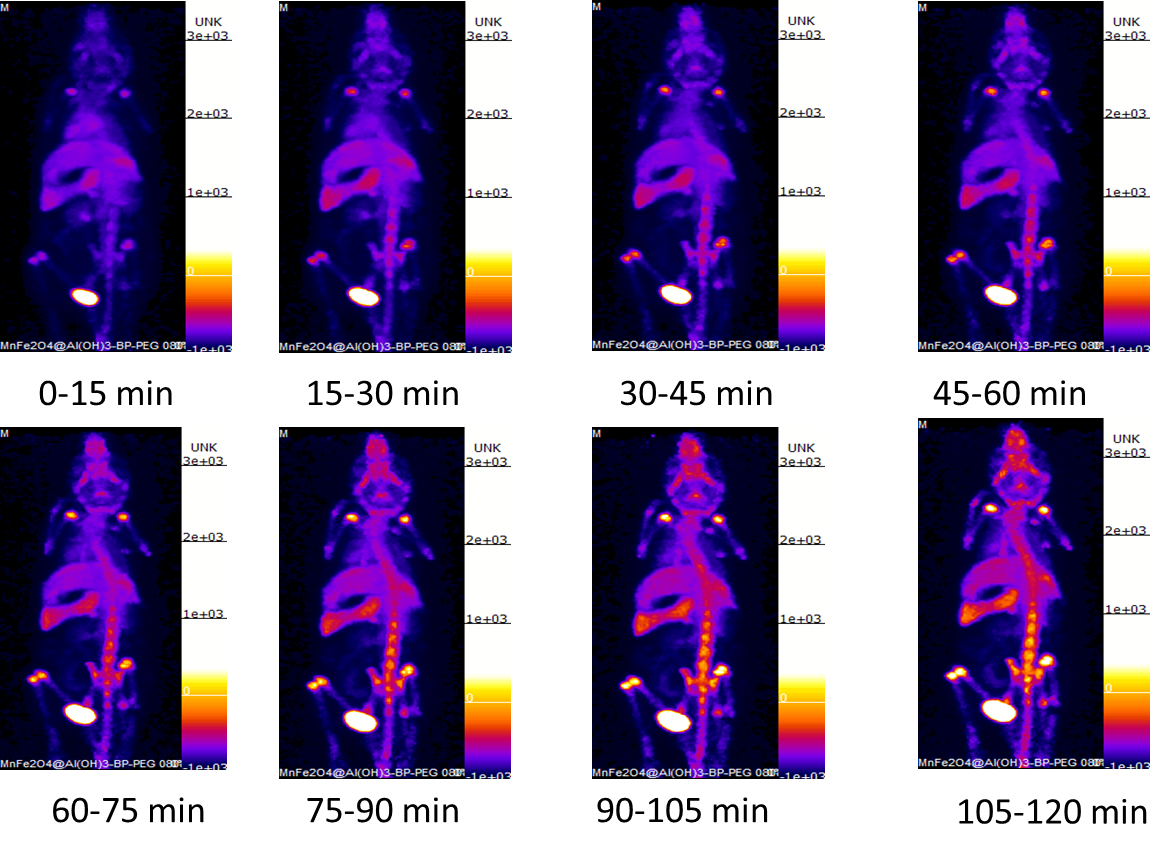
**

**Figure S9.** *In vivo* PET/CT images of a normal young C57BL/6 mouse using ^18^F radiolabelled ***1***: **a)** 0-15 minutes; **b)** 15-30 minutes; **c)** 30-45 minutes; **d)** 45-60 minutes; **e)** 60-75 minutes; **f)** 75-90 minutes; **g)** 90-105 minutes; and **h)** 105-120 minutes. NPs ***1*** obtained by a slow hydrolysis process has a better in vivo stability than NP ***3*** synthesised by a quick hydrolysis (Fig. S8), which is in consistent with the in vitro studies.


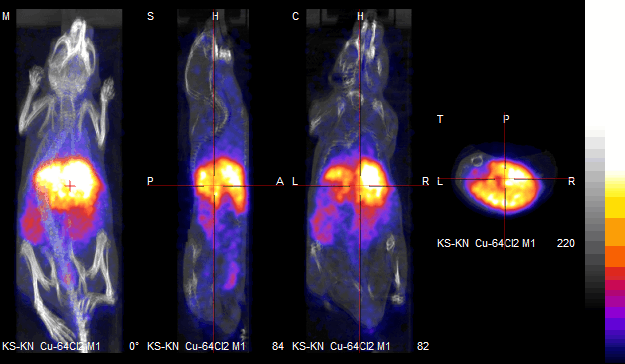


**Figure S10.** *In vivo* PET/CT images of a normal young C57BL/6 mouse using ^64^CuCl_2_ solution (0-30 minutes).

**
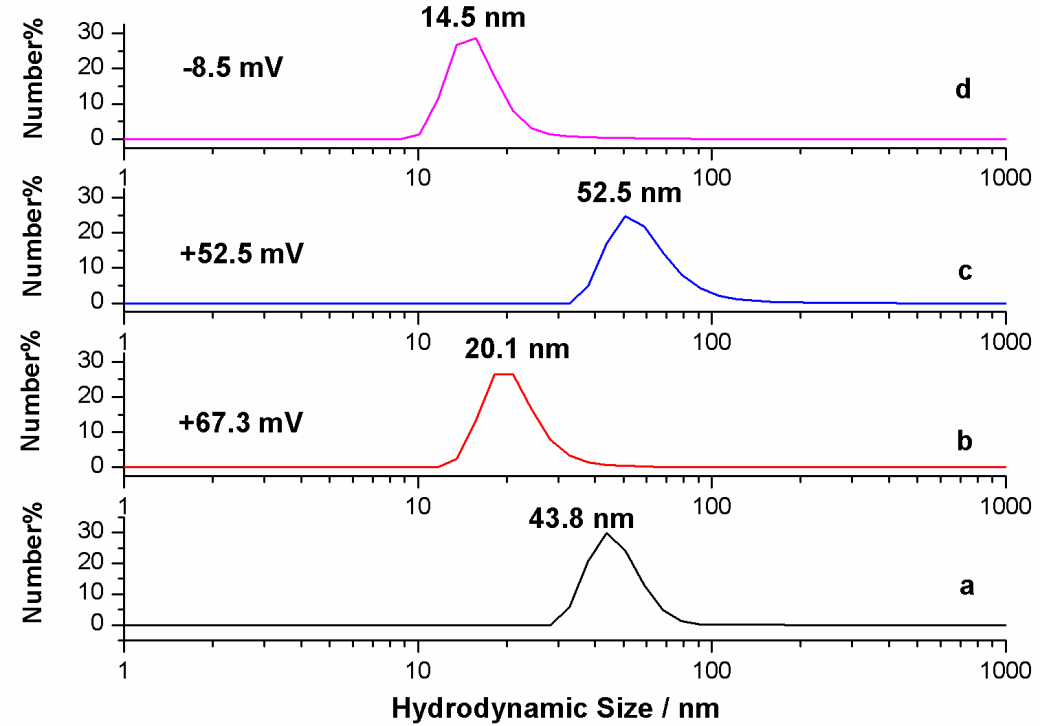
**

**Figure S11.** DLS size distribution of NPs: **a)** Fe_3_O_4_ NPs in hexane; **b)** pre-washed NPs ***3*** in water; **c)** post-washed NPs ***3*** in water; and **d)** conjugates of post washed NPs ***3*** and BP-PEG (10K Da). [***3***] = [Fe_3_O_4_] ≈ 1 mg/L. Zeta potential was measured in neutral aqueous solution with a pH value ≈ 7.
